# Mobitz II Atrioventricular Block Following Intracardiac Radiation to the Right Ventricular Outflow Tract

**DOI:** 10.7759/cureus.40731

**Published:** 2023-06-21

**Authors:** Jackson B Troxel, Grant R Conner

**Affiliations:** 1 Internal Medicine, NEA Baptist Memorial Hospital, Jonesboro, USA

**Keywords:** cardiovascular disease, radiotherapy (rt), cardiac conduction defects, cardiac electrophysiology, mri cardiac, cardiac pacemaker

## Abstract

Cardiac complications from mediastinal radiotherapy are much more prevalent than in years past and are becoming a significant cause of morbidity and mortality in these patients following treatment. We describe a patient with metastatic lung adenosquamous carcinoma extending to the right ventricular outflow tract who would develop a Mobitz type II atrioventricular block following intracardiac radiation therapy requiring permanent pacemaker placement.

## Introduction

Radiation-induced heart disease (RIHD) describes a spectrum of pathologies that can present in patients who receive mediastinal radiation therapy (RT) and includes pericarditis, cardiomyopathy, coronary artery disease (CAD), valvular heart disease, and cardiac conduction abnormalities. This condition is thought to have been previously underreported, as the long-term survival of patients with intrathoracic tumors has historically been low, but more research has been published in recent years due to improved survival rates of patients who have received mediastinal RT. While technological advances have greatly reduced the morbidity associated with RIHD, they have not been found to eliminate the risk of developing RIHD following RT [1].

RIHD can be further classified into acute and chronic forms. The acute forms, which can present immediately after receiving RT, are acute pericarditis, which is thought to be the earliest form of RIHD and acute conduction system abnormalities. The chronic forms of RIHD, typically 10-15 years following radiation, are chronic pericarditis, ischemic heart disease, cardiomyopathy, heart failure, valvular heart disease, and chronic conduction system abnormalities [2].

The pathogenesis of RIHD is thought to be multifactorial and largely due to increased oxidative stress and free radical formation. In the acute phase, endothelial cellular injury is thought to be the primary cause of radiation injury to cardiac tissue and leads to the production of proinflammatory molecules, including prostaglandins, prostacyclins, thromboxanes, and leukotrienes which lead to tissue necrosis and autophagy of cardiac tissue. The progression of chronic RIHD is thought to be largely mediated by increased expression and activity of growth factors, such as TGF-β, that promote cardiac hypertrophy, tissue fibrosis, and increased collagen deposition and stenosis in the lumen of coronary vasculature [2].

## Case presentation

**Figure 1:**
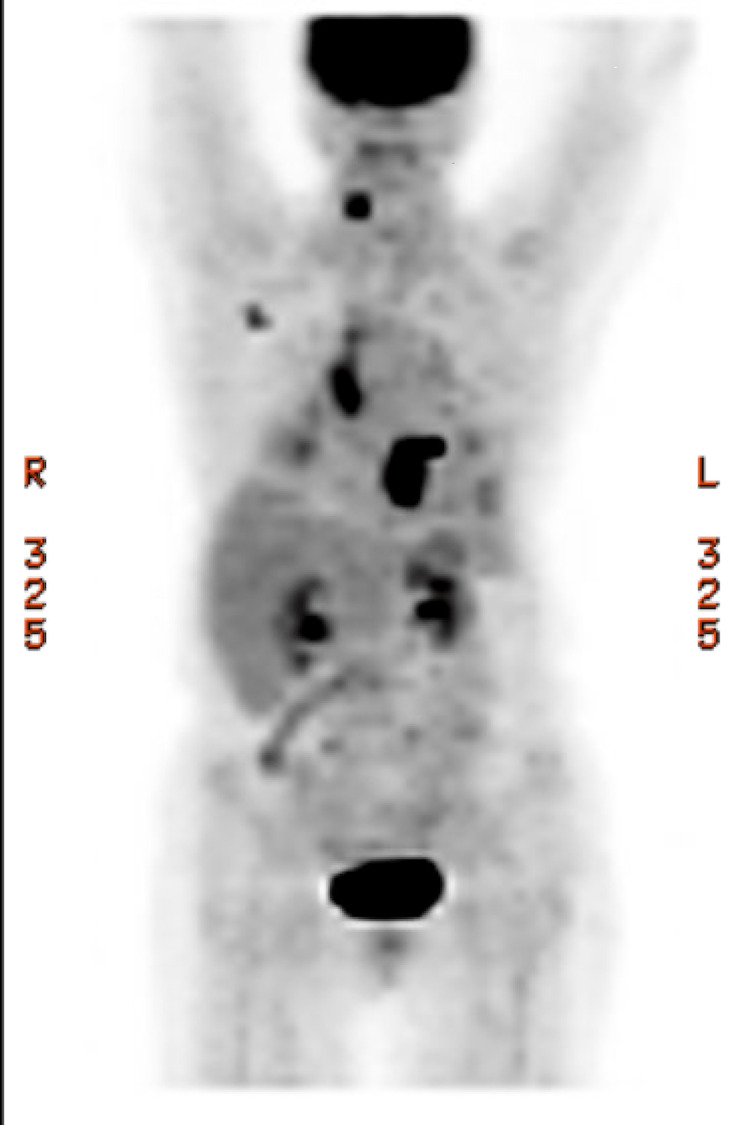
PET scan PET scan showing increased activity over the area of the RV. RV: right ventricle

**Figure 2:**
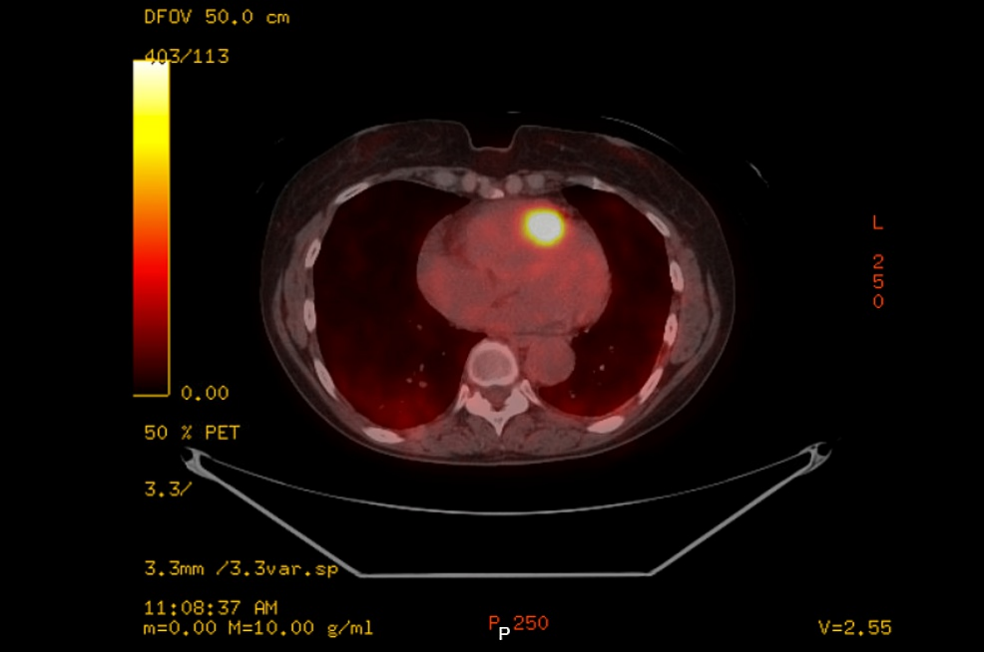
PET/CT scan PET/CT fused axial image showing increased fluorodeoxyglucose uptake by the RV mass. RV: right ventricle

A 68-year-old female with a history of metastatic adenosquamous carcinoma of the lung with secondary squamous cell carcinoma of the right ventricular outflow tract (RVOT) presented with a three-week history of generalized weakness, worsening dyspnea with minimal exertion, and palpitations. A review of previous records revealed she had a complex oncological history with periods of remission complicated by the progression of her malignancy. Approximately eight months before presentation, she underwent a restaging positron emission tomography/computed tomography (PET/CT) scan that identified widespread thoracic metastases with a nodule in the right ventricle (RV) concerning for a cardiac metastasis (Figures 1-2).

**Figure 3:**
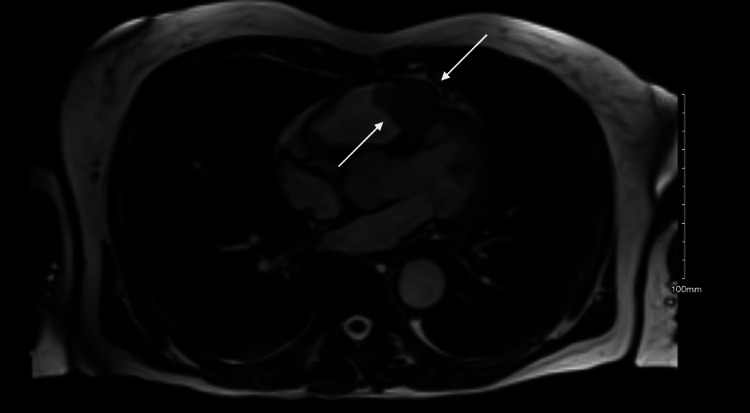
Cardiac MRI Axial view from cardiac MRI showing RV with mass identified with white arrows. RV: right ventricle

**Figure 4:**
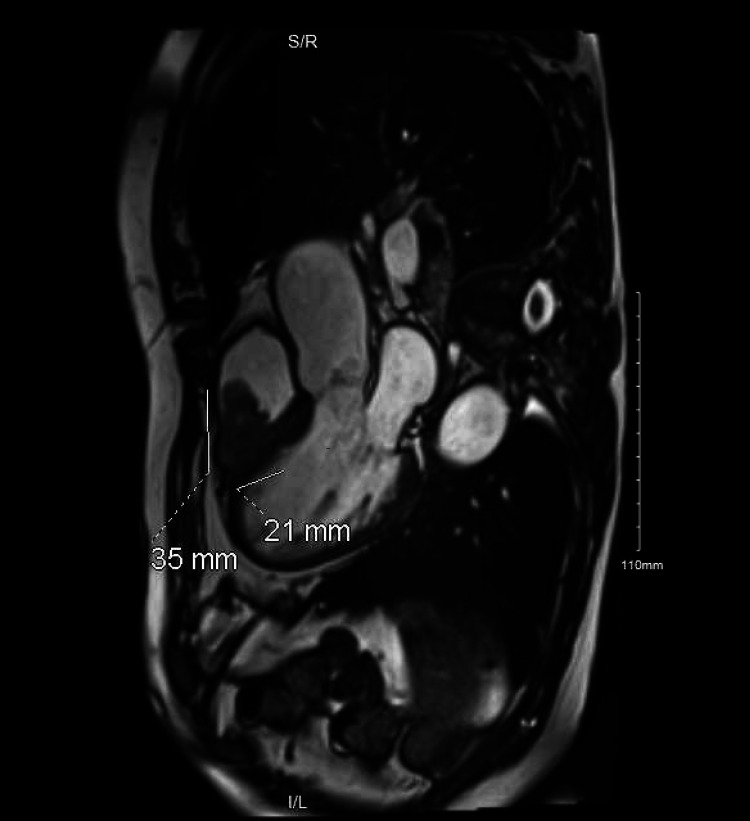
Cardiac MRI (2) Sagittal view from cardiac MRI showing RV mass with measurements. RV: right ventricle

Cardiac magnetic resonance imaging (MRI) was then performed following the PET/CT that identified a 21x21x35 mm mass in the RVOT within approximately 1.6 cm of the pulmonic valve and encroachment on the interventricular septum (Figures 3-4).

**Figure 5:**
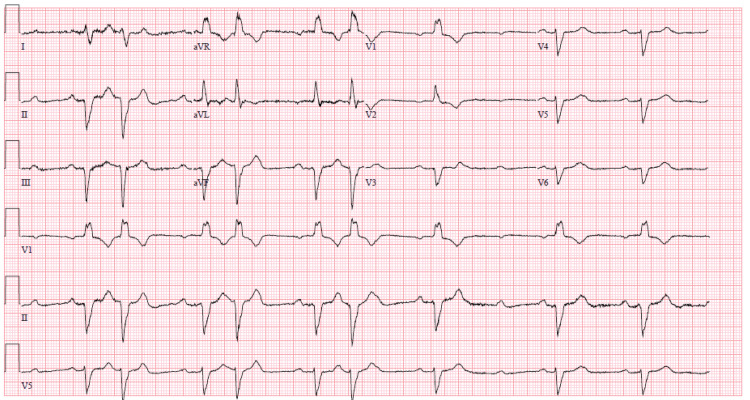
Electrocardiogram ECG with Mobitz II AV block

**Figure 6:**
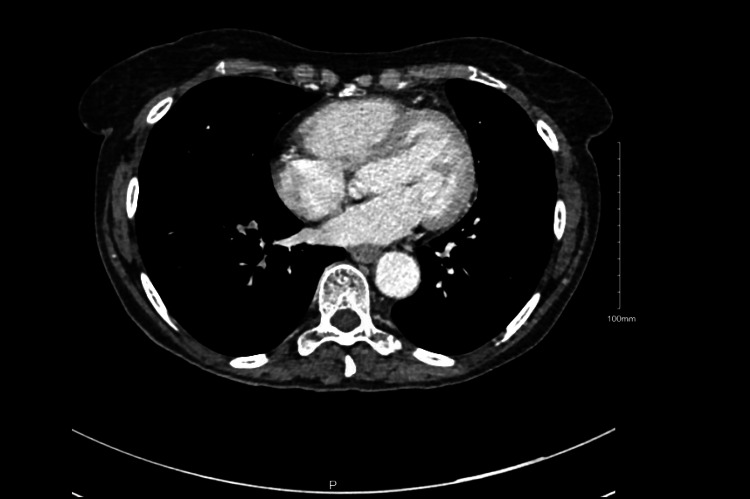
CT scan CT scan from admission with a complete radiographic response of the RV mass following radiotherapy. RV: right ventricle

Given these findings, she was started on palliative intracardiac radiotherapy and would receive eight total treatments with the last approximately two months prior to her presentation. On initial evaluation in the emergency department, an electrocardiogram (ECG) was obtained and revealed a new second-degree atrioventricular (AV) block type II with 2:1 conduction with premature supraventricular contractions and a right bundle branch block (Figure 5). CT scan would show a complete radiographic response of the mass in the RV (Figure 6).

Cardiology was consulted on admission, and she underwent placement of a dual chamber pacemaker with the RV lead implanted in the septum. Following the procedure, her symptoms rapidly improved, and she was able to be discharged home. Unfortunately, this patient’s malignancy would worsen and spread further throughout her thorax, and she ultimately passed away from complications that arose from the progression of her disease.

## Discussion

Approximately 75 % of patients who receive radiation therapy to the mediastinum will have an ECG abnormality following radiation treatment, and conduction abnormalities have been noted with an overall incidence rate of 4-5% [3]. The proposed etiology for developing electrophysiological abnormalities is decreased microvessel density, chronic hypoxia, and compensatory hypertrophy that lead to fibrotic lesions [2]. There have been new protocols formulated to shield the heart from unintended exposure in an attempt to decrease the likelihood of developing cardiac conduction abnormalities following RT, as well as the development of newer technology that allows for a more precise direction of the radiation beam to limit exposure to the heart [4]. Current guidelines recommend evaluating patients for heart failure, valvular disease, and CAD 5 years after they receive mediastinal radiation and then at 5-year intervals, even if they are asymptomatic, but currently, there is no recommendation to screen these patients for conduction abnormalities [5]. Given the high incidence of cardiac conduction abnormalities in patients who have received mediastinal RT, physicians must be aware of and monitor for these in the chronic management of these patients.

There is currently no medical therapy in use to prevent or treat RIHD, but a recent study has found that toll-like receptor 4 (TLR4) is upregulated in RIHD and leads to increased oxidative stress, ECG abnormalities, and abnormal tissue fibrosis and remodeling and postulated that use of a TLR4 inhibitor might alleviate RIHD [6]. The significance of toll-like receptors in RIHD was also noted in a study that found dexrazoxane (DZR), a medication currently used to prevent cardiotoxicity in patients receiving an anthracycline, significantly reduced expression of IKBKE, MAP3K8, NFKBIA, and TLR5, four genes involved in the toll-like receptor pathway, in irradiated cardiac tissue from rat models that received DZR in combination with RT compared to RT alone [7].

## Conclusions

This case presents an uncommon cause of a common cardiovascular condition and highlights the importance of being aware of and monitoring potential cardiac complications following mediastinal RT. Given the advancements in the treatment of various cancers and the improved survival rates in these patients, it is likely that RIHD will become much more prevalent in the coming years. Due to this, this case serves as a reminder for physicians caring for patients who have received mediastinal radiation to monitor for the long-term adverse effects that may present in the months to years following treatment.
